# Repeat cytoreductive surgery with or without intraperitoneal chemotherapy for recurrent epithelial appendiceal neoplasms

**DOI:** 10.1002/bjs5.50262

**Published:** 2020-02-05

**Authors:** J. B. Karpes, J. D. Lansom, M. Alshahrani, R. Parikh, R. Shamavonian, N. A. Alzahrani, W. Liauw, D. L. Morris

**Affiliations:** ^1^ Liver and Peritonectomy Unit St George Hospital Sydney New South Wales Australia; ^2^ Cancer Care Clinic St George Hospital Sydney New South Wales Australia; ^3^ St George and Sutherland Clinical School University of New South Wales Sydney New South Wales Australia; ^4^ College of Medicine Al Imam Mohammad Ibn Saud Islamic University Riyadh Saudi Arabia

## Abstract

**Background:**

With recurrence rates after primary cytoreductive surgery (CRS) in excess of 50 per cent, repeat CRS is being performed increasingly, but survival outcomes have not been reported widely. This study examined the outcomes following repeat CRS for appendiceal cancer with peritoneal surface malignancy (PSM), and evaluated its feasibility and safety.

**Methods:**

A retrospective cohort of patients who had surgery between 1996 and 2018 were analysed. Patients who underwent a single CRS procedure with or without heated intraperitoneal chemotherapy (HIPEC) were compared with those who had multiple procedures with or without HIPEC. Perioperative morbidity and survival outcomes were analysed.

**Results:**

Some 462 patients were reviewed, 102 of whom had repeat procedures. For high‐grade tumours, patients who had a single CRS procedure had significantly reduced overall survival (OS) compared with those who had repeat CRS (55·6 *versus* 90·7 months respectively; *P* = 0·016). For low‐grade tumours, there was no difference in OS (*P* = 0·153). When patients who had a single procedure were compared with those who had multiple procedures, there was no significant difference in major morbidity (*P* = 0·441) or in‐hospital mortality (*P* = 0·080). For multiple procedures, no differences were found in major morbidity (*P* = 0·262) or in‐hospital mortality (*P* = 0·502) when the first procedure was compared with the second. For low‐grade cancers, the peritoneal carcinomatosis index was a significant prognostic factor for OS (hazard ratio (HR) 1·11, 95 per cent c.i. 1·05 to 1·17; *P* < 0·001), whereas for high‐grade cancers repeat CRS (HR 0·57, 0·33 to 0·95; *P* = 0·033), complete cytoreduction score (HR 1·55, 1·01 to 2·40; *P* = 0·046) and presence of signet ring cells (HR 2·77, 1·78 to 4·30; *P* < 0·001) were all significant indicators of long‐term survival.

**Conclusion:**

In selected patients presenting with PSM from epithelial appendiceal neoplasms, repeat CRS performed in high‐volume centres could provide survival benefits.

## Graphical abstract

Cytoreductive surgery (CRS) has been established as the standard for the management of peritoneal surface malignancy (PSM); however, the utility of repeat CRS for recurrent epithelial appendiceal neoplasms has not been explored widely. A large retrospective cohort analysis was performed of patients with PSM of appendiceal origin, evaluating the short‐ and long‐term outcomes. The findings showed that, with careful patient selection, repeat CRS for PSM of appendiceal origin can result in long‐term survival with acceptable morbidity.

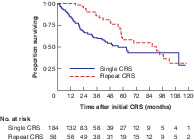

Citoreductive surgery in peritoneal malignancy fro appendiceal neoplasm

## Abstract (es)

**Antecedentes:**

En países de bajos y medianos ingresos (*low‐ and middle‐income countries*, LMIC) hay que desarrollar estrategias de futuro para incrementar la disponibilidad de equipos quirúrgicos, adquisición, capacitación, uso, mantenimiento y complicaciones relacionadas con las unidades electroquirúrgicas (*electrosurgical unit*, ESU) y los equipos de laparoscopia.

**Métodos:**

Se realizó una encuesta entre los cirujanos que asistieron a la reunión anual del Colegio de Cirujanos de África Oriental, Central y Meridional (*College Of Surgeons of East, Central and Southern Africa*, COSECSA) en diciembre de 2017 y a la reunión anual de la Sociedad Quirúrgica de Kenia (*Surgical Society of Kenya*, SSK) en marzo de 2018. Se encuestaron también a los técnicos de equipos biomédicos (*Biomedical Equipment Technicians*, BMET) y se recopilaron los registros de mantenimiento en Kenia entre febrero y marzo de 2018.

**Resultados:**

Participaron 80 sujetos, 59 cirujanos de 11 países africanos y 21 BMET de Kenia. Se recopilaron 36 registros de mantenimiento. Todos los cirujanos de COSECSA y SSK disponían de ESU, pero menos de la mitad (49%) disponían de equipos de laparoscopia. Como principales problemas se detectaron la reutilización de accesorios desechables en las ESU y las dificultades para disponer de CO2. Más de las tres cuartas partes (78%) de los cirujanos contaban con equipos de mantenimiento para las ESU, pero solo el 59% disponía de mantenimiento para los equipos de laparoscopia en su centro.

**Conclusión:**

A pesar de la disponibilidad de equipos quirúrgicos, en estos LMIC se detectaron serias dificultades en su mantenimiento, hecho que limita la implementación de la cirugía abierta y laparoscópica.

## Introduction

Primary epithelial appendiceal neoplasms are rare primary malignancies of the gastrointestinal tract, accounting for less than 0·5 per cent of all gastrointestinal neoplasms^1^, ^2^, ^3^, and representing only 1 per cent of colorectal cancers^4^. Locoregional dissemination into the peritoneum is not uncommon, with appendiceal neoplasm being one of the main causes of peritoneal surface malignancy (PSM)^5^, ^6^.

The traditional approach to the management of appendiceal neoplasms with PSM was palliative debulking surgery, in which the main objective was symptom relief via removal of the primary tumour, omental mass and all free mucus, as well as extensive wiping of the peritoneal surfaces^7^, ^8^, ^9^, ^10^. Over the past three decades, there has been significant progress in the management of PSM. Cytoreductive surgery (CRS) with heated intraperitoneal chemotherapy (HIPEC) has been established as a combined surgical and oncological treatment for PSM with curative intent^7–9,11–14^, and is now accepted as the standard of care for selected patients with PSM from appendiceal neoplasms^15^, ^16^, ^17^, ^18^, ^19^, ^20^. Data from the last two decades show that CRS–HIPEC is associated with a marked improvement in long‐term survival, with reported 10‐year survival rates above 60 per cent^5^, ^9^, ^10^, ^16^, ^21^, ^22^, ^23^.

Despite the improved survival data, disease recurrence remains common, with estimates in excess of 50 per cent^12^. The median time to recurrence is 44 months in patients with appendiceal mucinous neoplasms^24^, ^25^, ^26^. In a past study^8^, disease progression was the only independent risk factor for reduced overall survival (OS) in patients with PSM from appendiceal neoplasms. Repeat CRS–HIPEC has been shown to be a viable treatment alternative for these patients^6^, ^12^, ^13^, ^27^. A large retrospective analysis^28^ found that patients who underwent repeat CRS had significantly higher 5‐year and overall survival rates than those who did not, with no significant difference in major morbidity or in‐hospital mortality. Other reports^9^, ^13^, ^17^, ^27^ have supported these findings, although the literature is still limited.

The objective of this study was to examine the short‐ and long‐term outcomes of patients undergoing repeat CRS with or without HIPEC as treatment for recurrent appendiceal neoplasm.

## Methods

Patients undergoing CRS–HIPEC for PSM from appendiceal neoplasms between January 1996 and July 2018 at St George Hospital in Sydney, Australia, were reviewed. The unit has experience in performing over 1300 cases of CRS–HIPEC for PSM from a variety of tumour origins, of which over 250 have been repeat procedures. All patients undergoing CRS with or without HIPEC for treatment of PSM from an appendiceal primary were identified.

The indication for CRS with or without HIPEC was assessed during a regular weekly multidisciplinary team (MDT) meeting that included surgical oncologists, medical oncologists, radiologists, cancer care nurses and research staff. Patients selected for a primary procedure were those with a histological diagnosis of PSM of epithelial appendiceal origin who had a good performance status (WHO performance status 2 or above).

Patients were selected for repeat CRS with or without HIPEC if they demonstrated evidence of recurrent peritoneal metastasis, identified by suspicious clinical examination findings, increased tumour markers and/or CT findings during standard follow‐up after the primary procedure or emergency presentation. CT was complemented by PET, which was used as an adjunct to rule out extra‐abdominal metastatic disease (an exclusion criterion). Patients with isolated intra‐abdominal recurrence who were deemed to have resectable disease, and were fit for further major surgery, were discussed at the weekly MDT meeting and considered for repeat CRS–HIPEC^28^.

Patients who underwent a single CRS procedure with or without HIPEC were compared with those who had multiple CRS procedures with or without HIPEC. In addition, patients who underwent multiple CRS procedures were further investigated for comparison of short‐ and long‐term outcomes between the first and second procedure. Subgroup analyses were performed according to histopathological grade.

The study was approved by St George Hospital's Human Research Ethics Committee, which waived the requirement for informed consent from patients as the data collected were non‐identified, thereby preserving patient anonymity.

### Histopathology

Epithelial appendiceal neoplasms were grouped based on tumour grade, in accordance with the consensus for classification of appendiceal neoplasia outlined by the Peritoneal Surface Oncology Group International at their World Congress in Berlin, 2012^29^. Low grade included tumours that demonstrated only ‘pushing invasion’ into the surrounding mucosa, alternatively referred to as diffuse peritoneal adenomucinosis (DPAM). High grade was reserved for tumours with a desmoplastic response entailing infiltrative invasion, also known as peritoneal mucinous carcinomatosis (PMCA). Patients with signet ring cells (SRC) comprise a subgroup with high‐grade tumours.

### Preoperative management

All patients had standard preoperative workup, including history and physical examination, routine haematological and biochemistry blood tests (including tumour markers carcinoembryonic antigen, carbohydrate antigen (CA) 125 and CA19‐9), and contrast‐enhanced CT of the chest, abdomen and pelvis. Dedicated imaging of the liver and fluorodeoxyglucose‐PET was performed in patients with high‐grade tumours.

### Cytoreductive surgery

CRS was performed using Sugarbaker's technique^11^, with the aim of removing all macroscopic peritoneal disease. This frequently involved multivisceral resection and multiple peritonectomy procedures. All sites and volumes of residual disease following the procedure were recorded prospectively using the completeness of cytoreduction (CC) score. The volume of disease was recorded using the peritoneal carcinomatosis index (PCI)^30^.

### Intraperitoneal chemotherapy

After CRS, HIPEC was delivered at approximately 42°C using either oxaliplatin (350 mg/m^2^) over 30 min or mitomycin C (12·5 mg/m^2^) over 90 min, depending on tumour type. Early postoperative intraperitoneal chemotherapy (EPIC) was used in patients with low‐grade tumours^31^. EPIC was also used, for example, when HIPEC was unavailable (emergency cases). The agent used was 5‐fluorouracil (650 mg/m^2^) for 2–6 days after surgery in the ICU.

### Data and outcome measures

Demographic characteristics, operative details, PCI and CC scores were reviewed for each patient. Outcome measures included perioperative morbidity and mortality, and survival. Perioperative morbidity was classified using the Clavien–Dindo classification of surgical complications^32^. Hospital mortality was defined as death during the same hospital admission as that for CRS. Follow‐up was conducted at 3‐monthly intervals for the first 12 months and 6‐monthly thereafter, until the last date of contact or death. Routine follow‐up entailed clinical examination, measurement of serum tumour markers, and CT of the chest, abdomen and pelvis. OS was calculated from the initial procedure to the last date of contact or death.

### Statistical analysis

Parametric continuous data were compared using the two‐sample *t* test and non‐parametric continuous data with the Mann–Whitney *U* test. Frequencies between groups were compared using the χ^2^ test or Fisher's exact test, as appropriate. Survival analysis was performed using the Kaplan–Meier method. Univariable hazard ratios (HRs) were calculated for categorical variables with the log rank test, and Cox regression for continuous variables. A multivariable model was created using Cox regression. *P* < 0·050 was considered statistically significant. All statistical analyses were performed using Stata® software version 15 (StataCorp, College Station, Texas, USA).

## Results

Between January 1996 and July 2018, 462 patients underwent primary CRS with or without HIPEC for PSM of appendiceal origin. Of these 462 patients, 102 had repeat procedures. Thirty patients underwent multiple repeat procedures, with ten patients having three repeats, four having four repeats, and two patients having five repeat procedures in total. There were 215 men (46·5 per cent) and 247 women (53·5 per cent). Of the total cohort, 220 patients (47·6 per cent) had low‐grade and 242 (52·4 per cent) had high‐grade tumours. Of the 242 patients with high‐grade tumours, SRC were found in 75 patients, 15 of whom were undergoing repeat procedures.

### Comparison of initial procedures for the whole cohort: single *versus* multiple cytoreductive surgery

*Table* 1 provides a comparison of the characteristics of the 360 patients who had only a single CRS–HIPEC procedure and the 102 who underwent multiple procedures. Median PCI score was lower in the single‐procedure group compared with that in patients who had multiple procedures (24 *versus* 29·5; *P* < 0·001). Patients who underwent multiple procedures were younger at the time of their initial procedure than those who had only a single procedure (51·1 *versus* 55·7 years respectively; *P* = 0·001).

**Table 1:** Characteristics of patients undergoing one or multiple cytoreductive surgery–heated intraperitoneal chemotherapy procedures at the time of the first procedure

	Single CRS–HIPEC (*n* = 360)	Multiple CRS–HIPEC (*n* = 102)	*P* †
**Mean age (years)**	55·7	51·1	0·001§
**Sex ratio (M** : **F)**	168 : 192	47 : 55	0·916¶
**Chemotherapy**			
HIPEC alone	160 (44·4)	33 (32·4)	0·029
HIPEC + EPIC	187 (51·9)	60 (58·8)	0·219
EPIC alone	10 (2·8)	4 (3·9)	0·521‡
**Histopathology**			0·305¶
Low grade	176 (48·9%)	44 (43·1)	
High grade	184 (51·1)	58 (56·9)	
**PCI score** *	24 (12–33)	29·5 (20–36)	< 0·001#
**CC score**			0·048
0–1	348 (96·7)	94 (92·2)	
2–3	12 (3·3)	8 (7·8)	
**Interval between procedures (months)** *		23·8 (11–33)	

Values in parentheses are percentages unless indicated otherwise;

* values are median (i.q.r.). CRS, cytoreductive surgery; HIPEC, heated intraperitoneal chemotherapy; EPIC, early postoperative intraperitoneal chemotherapy; PCI, peritoneal carcinomatosis index; CC, completeness of cytoreduction.

† χ^2^ test, except

‡ Fisher's exact test,

§ two‐sample *t* test,

¶ two‐sample test of proportions and

# Mann–Whitney *U* test.

In both groups, complete cytoreduction (CC 0–1) was achieved in the majority of patients, with 348 patients (96·7 per cent) in the single‐procedure group and 94 (92·2 per cent) in the multiple‐procedures group achieving macroscopic tumour clearance. The majority of patients in both groups received intraperitoneal chemotherapy (IPC). In the single‐procedure group, 357 patients (99·2 per cent) received HIPEC and/or EPIC. In the multiple‐procedures group, a total of 97 patients (95·1 per cent) had HIPEC and/or EPIC (χ^2^ = 7·7, *P* = 0·005).

#### *Perioperative outcomes*

*Table* 2 summarizes perioperative outcomes for patients who had only a single procedure *versus* those who had multiple procedures. There were significant differences in mean duration of surgery (533 min for single CRS *versus* 591 min for multiple CRS; *P* = 0·012) and mean number of packed red blood cells (RBC) transfused (4 *versus* 6 units respectively; *P* < 0·001). There was no significant difference in high‐dependency unit (HDU)/ICU length of stay (LOS) (*P* = 0·157), total hospital LOS (*P* = 0·165), major morbidity (*P* = 0·441) or in‐hospital mortality (*P* = 0·080).

**Table 2:** Perioperative outcomes for patients undergoing one or multiple cytoreductive surgery–heated intraperitoneal chemotherapy procedures at the time of the first procedure

	Single CRS–HIPEC (*n* = 360)	Multiple CRS–HIPEC (*n* = 102)	*P* ‡
Duration of surgery (min)*	533(154)	591(145)	0·012§
Blood transfusion (units)†	4 (0–8)	6 (3–13)	< 0·001
HDU/ICU LOS (days)†	4 (4–9)	7 (5–10)	0·157
Total LOS (days)†	23 (16–35)	25 (19–34)	0·165
Grade III–IV morbidity	170 (47·2)	53 (52·0)	0·441¶
Hospital death	10 (2·8)	0 (0)	0·080#

Values in parentheses are percentages unless indicated otherwise;

* values are mean(s.d.) and

† median (i.q.r.). CRS, cytoreductive surgery; HIPEC, heated intraperitoneal chemotherapy; HDU, high‐dependency unit; LOS, length of stay.

‡ Mann–Whitney *U* test, except

§ two‐sample *t* test,

¶ χ^2^ test and

# Fisher's exact test.

#### *Survival outcomes*

**Figure 1:**
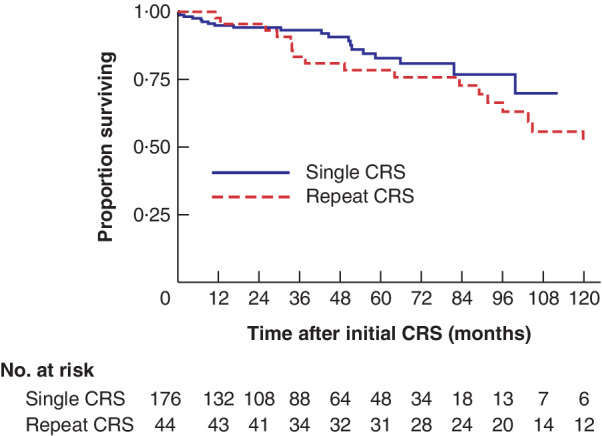
Kaplan–Meier analysis comparing overall survival in patients with low‐grade tumours undergoing initial or repeat cytoreductive surgery–heated intraperitoneal chemotherapy CRS, cytoreductive surgery. *P* = 0·153 (log rank test).

**Figure 2:**
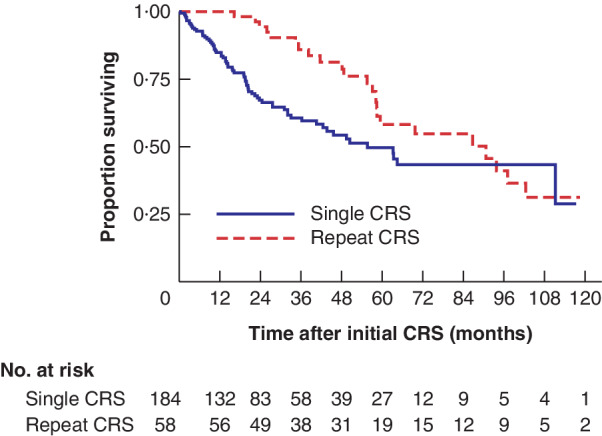
Kaplan–Meier analysis comparing overall survival in patients with high‐grade tumours undergoing initial or repeat cytoreductive surgery–heated intraperitoneal chemotherapy CRS, cytoreductive surgery. *P* = 0·016 (log rank test).

*Figs* 1 and 2 show the Kaplan–Meier curves for both low‐grade (DPAM) and high‐grade (PMCA) tumours OS in patients who had only a single procedure compared with that in patients having multiple procedures. For high‐grade tumours, there was a significant difference in OS from the time of first CRS: median OS 55·6 (95 per cent c.i. 40·5 to not reached (n.r.)) months in the single‐CRS group *versus* 90·7 (58·4 to 102·5) months in the multiple CRS group (*P* = 0·016) (*Fig*. 2). There was no difference in OS between the two groups for low‐grade tumours (248·7 (99·7 to n.r.) *versus* 125·5 (91·6 to n.r.) months respectively; *P* = 0·153) (*Fig*. 1).

For low‐grade tumours, 3‐ and 5‐year survival rates were better for the single‐CRS group than for the multiple‐CRS group (3 years: 93·2 *versus* 83·4 per cent respectively; 5 years: 82·8 *versus* 78·4 per cent). For high‐grade tumours, 3‐ and 5‐year survival rates were higher in the multiple‐CRS group than in the single‐CRS group (3 years: 85·9 *versus* 60·6 per cent respectively; 5 years: 58·2 *versus* 49·6 per cent).

#### *Multivariable analysis*

Univariable and multivariable analyses were performed to assess the prognostic significance of repeat CRS, age, PCI and CC scores, and presence of SRC for OS. For low‐grade tumours, only PCI score was a significant prognostic factor for OS (HR 1·11, 95 per cent c.i. 1·05 to 1·17; *P* < 0·001) (*Table* 3). For high‐grade tumours, repeat CRS (HR 0·57, 0·33 to 0·95; *P* = 0·033), CC score (HR 1·55, 1·01 to 2·40; *P* = 0·046) and presence of SRC (HR 2·77, 1·78 to 4·30; *P* < 0·001) remained as significant prognostic factors for OS (*Table* 4).

**Table 3:** Univariable and multivariable analysis of prognostic factors for overall survival in patients with low‐grade epithelial appendiceal neoplasms

	Univariable analysis		Multivariable analysis	
	Hazard ratio	*P*	Hazard ratio	*P*
Repeat CRS	1·62 (0·83, 3·15)	0·155	1·11 (0·54, 2·29)	0·773
Age	1·01 (0·99, 1·04)	0·196	1·02 (0·99, 1·05)	0·192
PCI score	1·12 (1·07, 1·18)	< 0·001	1·11 (1·05, 1·17)	< 0·001
CC score	2·62 (1·70, 4·03)	< 0·001	1·42 (0·82, 2·48)	0·206

Values in parentheses are 95 per cent confidence intervals. CRS, cytoreductive surgery; PCI, peritoneal carcinomatosis index; CC, completeness of cytoreduction.

**Table 4:** Univariable and multivariable analysis of prognostic factors for overall survival in patients with high‐grade epithelial appendiceal neoplasms

	Univariable analysis		Multivariable analysis	
	Hazard ratio	*P*	Hazard ratio	*P*
Repeat CRS	0·58 (0·35, 0·90)	0·017	0·57 (0·33, 0·95)	0·033
Age	1·01 (0·99, 1·03)	0·234	1·01 (0·99, 1·03)	0·569
PCI score	1·02 (0·99, 1·03)	0·086	1·01 (0·99, 1·03)	0·413
CC score	1·77 (1·25, 2·50)	0·001	1·55 (1·01, 2·40)	0·046
Signet ring cells	2·89 (1·87, 4·48)	< 0·001	2·77 (1·78, 4·30)	< 0·001

Values in parentheses are 95 per cent confidence intervals. CRS, cytoreductive surgery; PCI, peritoneal carcinomatosis index; CC, completeness of cytoreduction.

### Comparison of initial *versus* repeat cytoreductive surgery in patients undergoing multiple procedures

The median interval between the initial and repeat procedure in patients who had multiple CRS–HIPEC procedures was 23·8 (i.q.r. 11–33) months (*Table* 1). There was a difference in the PCI score for the initial procedure compared with the second procedure (median 29·5 (i.q.r. 20–36) *versus* 15 (7–26) respectively; *P* < 0·001). Complete cytoreduction was achieved in the majority of patients for both the initial and repeat procedures: 94 patients (92·2 per cent) for the initial procedure and 90 (88·2 per cent) for the repeat procedure, with no significant difference between the groups (*P* = 0·346) (*Table* 5).

**Table 5:** Perioperative outcomes for first and second cytoreductive surgery–heated intraperitoneal chemotherapy procedures in 102 patients who had a repeat procedure

	First CRS–HIPEC	Second CRS–HIPEC	*P* §
PCI score†	29·5 (20–36)	15 (7–26)	< 0·001
CC score 0–1	94 (92·2)	90 (88·2)	0·346#
Duration of surgery (min)*, ‡	591(145) (*n* = 54)	522(149) (*n* = 72)	0·010¶
Blood transfusion (units)†	6 (3–13)	2 (0–6)	< 0·001
HDU/ICU LOS (days)†	7 (5–10)	7 (4–9)	0·224
Total LOS (days)†	25 (19–34)	22 (15·5–35·5)	0·104
Grade III–IV morbidity	53 (52·0)	45 (44·1)	0·262#
Hospital death	0	1	0·502**

Values in parentheses are percentages unless indicated otherwise;

* values are mean(s.d.) and

† median (i.q.r.). CRS, cytoreductive surgery; HIPEC, heated intraperitoneal chemotherapy; PCI, peritoneal carcinomatosis index; CC, completeness of cytoreduction; HDU, high‐dependency unit; LOS, length of stay.

‡ Duration of surgery was not recorded before 2009.

§ Mann–Whitney *U* test, except

¶ two‐sample *t* test,

# χ^2^ test and

** Fisher's exact test.

The majority of patients received IPC in both first and second CRS procedures. For the initial CRS, 97 of the 102 patients received IPC, with 33 having HIPEC only and four only EPIC. For the repeat procedure, 98 patients received IPC, with 49 having HIPEC only and three only EPIC.

#### *Perioperative outcomes*

*Table* 5 shows the perioperative outcomes for patients who had multiple procedures. There was a significant difference for mean duration of surgery (591 min for initial CRS *versus* 522 min for repeat CRS; *P* = 0·010) and mean number of packed RBC transfused (6 *versus* 2 units respectively; *P* < 0·001). There was no significant difference in HDU/ICU LOS (*P* = 0·224), total hospital LOS (*P* = 0·104), major morbidity (*P* = 0·262) or in‐hospital mortality (*P* = 0·502).

#### *Survival outcomes*

**Figure 3:**
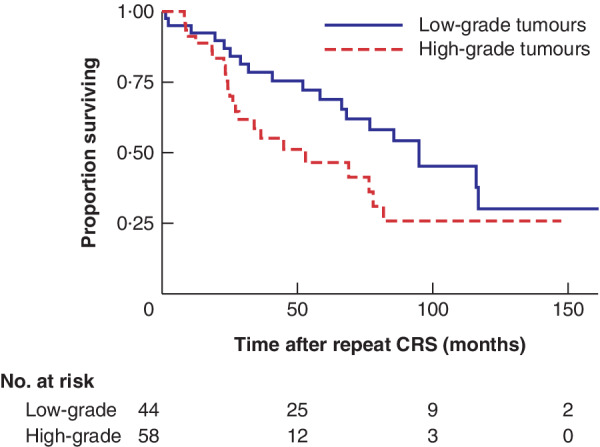
Kaplan–Meier analysis comparing overall survival in patients with low‐ and high‐grade tumours undergoing repeat cytoreductive surgery–heated intraperitoneal chemotherapy CRS, cytoreductive surgery. *P* = 0·054 (log rank test).

For low‐grade tumours, the 1‐, 3‐ and 5‐year OS rate after the initial CRS was 97·7, 83·4 and 78·4 per cent respectively, with rates after the repeat procedure of 92·4, 78·5 and 68·9 per cent. For high‐grade tumours, the 1‐, 3‐ and 5‐year OS rate after the initial CRS was 100, 85·9 and 58·2 per cent respectively, and 91·3, 58·6 and 46·5 per cent after the repeat procedure (*Fig*. 3).

## Discussion

In the past, PSM was considered an end‐stage disease. Since the introduction of CRS–HIPEC there have been significantly improved survival outcomes, with 5‐year survival rates of up to 74 per cent in patients with tumours of appendiceal origin^10^. However, disease recurrence after complete cytoreduction has been reported in up to 50 per cent of patients treated with CRS–HIPEC^9^. One study^7^ found that at least one‐third of patients experiencing disease progression after initial CRS–HIPEC could be considered as eligible candidates for further surgical intervention.

The present study has reported short‐ and long‐term outcomes after repeat CRS–HIPEC for a large series of patients with PSM of appendiceal origin. Few studies have been performed to investigate specifically repeat CRS–HIPEC for PSM of appendiceal origin, reporting outcomes on 98, 26 and 13 patients respectively^6^, ^7^, ^9^.

Patient selection for repeat CRS–HIPEC is crucial, and there are no formal criteria to draw upon. In the authors' centre, patient selection for repeat CRS is discussed at an MDT meeting. Patients with recurrent PSM of appendiceal origin are considered for repeat CRS if they have a good performance status, no extraperitoneal metastases and, generally, if the interval between operations is at least 12 months. High PCI score is not a contraindication to attempting repeat CRS–HIPEC if a complete cytoreduction can be achieved.

A past report^6^ documented OS rates following first CRS–HIPEC as 100, 83 and 54 per cent at 1, 3 and 5 years respectively, and 91, 53 and 34 per cent after the second procedure. Kitai and Yamanaka^9^ reported 5‐year survival rates after first and second CRS of 75·5 and 67·7 per cent respectively. The present results documented similar survival for patients with both low‐ and high‐grade tumours treated with multiple procedures. For low‐grade tumours, the 1‐, 3‐ and 5‐year OS rates were 97·7, 83·4 and 78·4 per cent respectively after the first procedure, and 92·4, 78·5 and 68·9 per cent after the second procedure. For high‐grade tumours, the respective rates were 100, 85·9 and 58·2 per cent after the first procedure, and 91·3, 58·6 and 46·5 per cent after the second procedure. Another study^6^ reported 5‐year survival rates for PMCA of 32 and 0 per cent after first and second CRS procedures respectively, whereas the respective 5‐year OS rates in the present study were 58·2 and 46·5 per cent. These findings demonstrate that grade of tumour should not, by itself, be a contraindication to repeat CRS–HIPEC.

An interesting development over the past decade in the management of recurrent pseudomyxoma peritonei (PMP) is modified multivisceral transplantation^33^. Reddy and colleagues^33^ asserted that recurrent PMP inevitably progresses to nutritional failure as a result of small bowel obstruction and fistulation, and proposed that, for patients with end‐stage PMP, CRS followed by modified multivisceral transplantation could prolong life. They reported on six patients who had this treatment between 2013 and 2016; four survived at review of 2, 4, 18 and 22 months after surgery, and two had died (on days 26 and 64). Despite promising survival data being reported for this new treatment modality, the extensive and severe complications that can ensue, including graft failure and death, highlight the positive significance of the improved survival outcomes being reported here, with relatively acceptable morbidity.

Previous studies^7^, ^12^ have shown that patients who undergo repeat CRS have improved outcomes compared with those who have only a single procedure. The present findings demonstrate improved OS in patients who have multiple procedures for recurrent PSM of high‐grade appendiceal origin in comparison with those who have only a single procedure (median 90·7 *versus* 55·6 months respectively; *P* = 0·016). This is promising for a tumour type that has been considered a negative predictor of survival in the past^34^. No significant difference in survival outcomes was documented between single or multiple CRS for low‐grade tumours, possibly because of their indolent tumour biology. One recent study^17^ demonstrated a lack of survival benefit in patients with low‐grade tumours who had repeat CRS *versus* those who did not. This was attributed to the excellent long‐term survival after the initial CRS for low‐grade tumours.

A systematic review^20^ found a morbidity rate of 12–52 per cent after CRS–HIPEC and a mortality rate of 0·9–5·8 per cent, whereas the present study observed major morbidity and mortality rates of 47·2 and 2·8 per cent respectively after a single procedure and 52·0 and 0 per cent following multiple procedures, with no significant difference whether comparing a single procedure with multiple procedures, or subsequent repeat procedures in the multiple CRS group. This demonstrates the safety of the CRS–HIPEC procedure.

This study is limited by its observational nature. Patient selection is critical in repeat CRS–HIPEC and, as this is a retrospective analysis, the lack of formal guidelines makes it difficult to derive any definitive selection criterion. Additionally, there is evidence that a learning curve exists for performing CRS–HIPEC^35^. It is likely that an additional learning curve may apply to the performance of repeat CRS–HIPEC procedures. Less experienced centres should consider referring to more experienced centres for repeat CRS–HIPEC. Another important limitation is the high volume of patients referred to the authors' centre from interstate and overseas. A consequence of this is missing information; in particular in the present data set, chemotherapy history was lacking.

With careful patient selection, repeat CRS–HIPEC can result in long‐term survival in a significant proportion of patients, with acceptable morbidity and mortality rates.

## Acknowledgements

J.B.K. and J.D.L. contributed equally to this article as first authors.

*Disclosure*: The authors declare no conflict of interest.
